# Supplementary figures and images for: Exploring bioactive compound origins: Profiling gene cluster signatures related to biosynthesis in microbiomes of Sof Umer Cave, Ethiopia
Source: PLoS One. 2025 Mar 6;20(3):e0315536. doi: 10.1371/journal.pone.0315536 (PMC11884727; doi:10.1371/journal.pone.0315536)

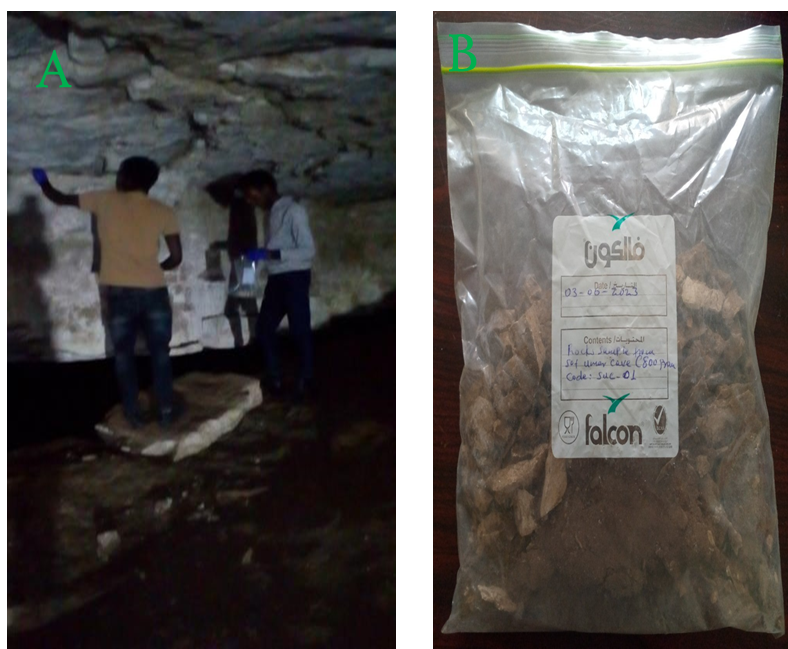


**S1 Fig 1A. Samples taken from the wall of Sof Umer Cave. 1B. Collected and homogenized samples.**

Supplement: S1 Fig — (DOCX) [file pone.0315536.s001.docx]

**S1 Fig 6. Putative biosynthesis-related gene cluster regions annotated by antiMASH.**


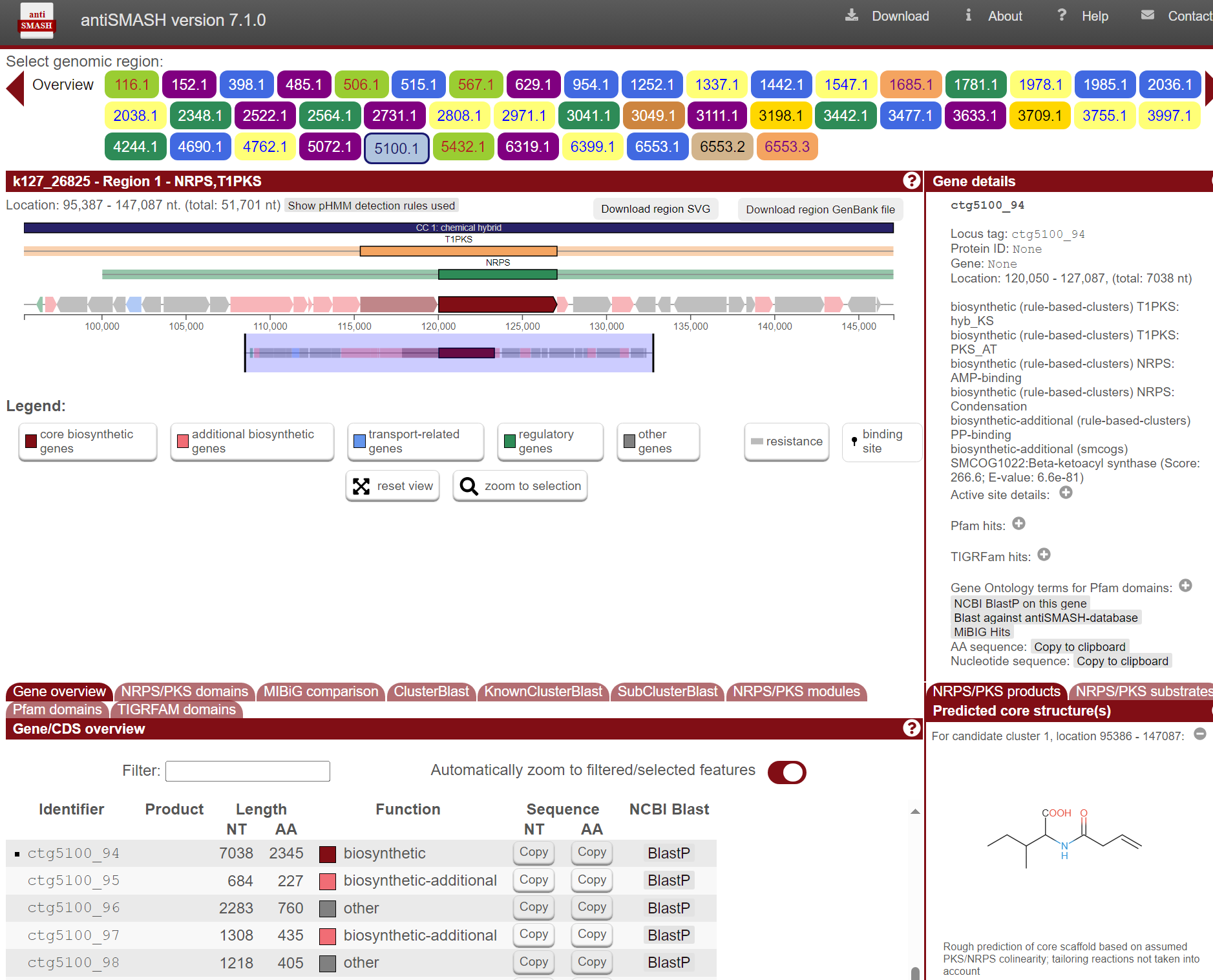

Supplement: S6 Fig — (DOCX) [file pone.0315536.s007.docx]
