# Supplementary material for: Exploring bioactive compound origins: Profiling gene cluster signatures related to biosynthesis in microbiomes of Sof Umer Cave, Ethiopia
Source: PLoS One. 2025 Mar 6;20(3):e0315536. doi: 10.1371/journal.pone.0315536 (PMC11884727; doi:10.1371/journal.pone.0315536)
Supplement: S2 Fig — (DOCX) [file pone.0315536.s002.docx]

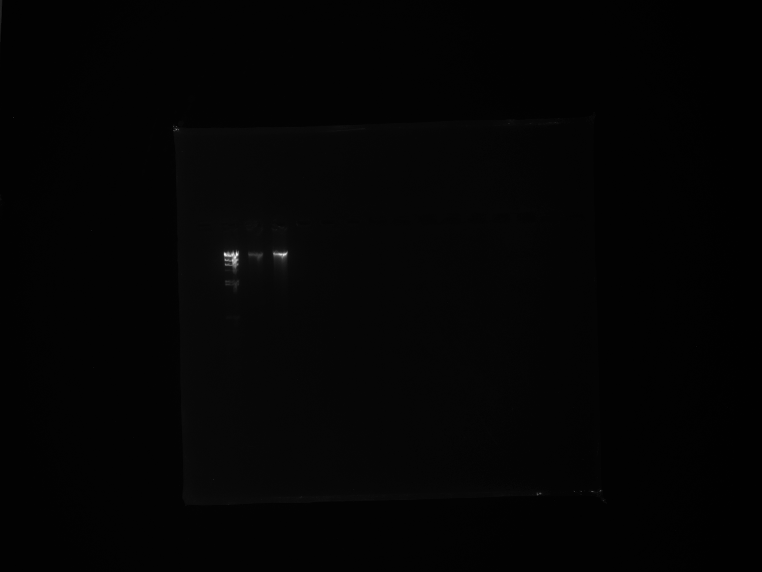

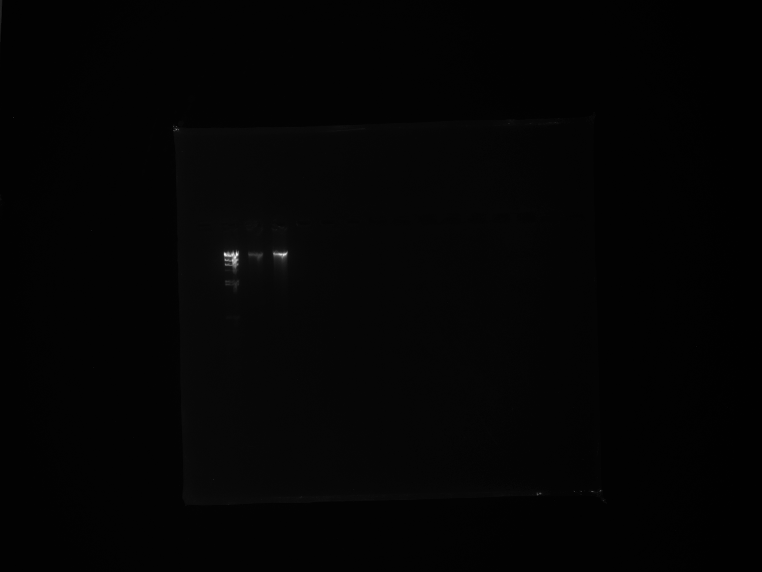


Metagenomic DNA

Lambda III

(23Kb)

R1

R1

A. Original Image B. Edited/Manipulated image

**S1 Fig 2. Agarose gel electrophoresis of metagenomic DNA extracted from rocks from Umer Cave.** Lane 1: Lambda III (Hind III double digest ladder), Lanes 2 and 3: metagenomic DNA extracted from rocks from Umer Cave.
