# Supplementary material for: Exploring bioactive compound origins: Profiling gene cluster signatures related to biosynthesis in microbiomes of Sof Umer Cave, Ethiopia
Source: PLoS One. 2025 Mar 6;20(3):e0315536. doi: 10.1371/journal.pone.0315536 (PMC11884727; doi:10.1371/journal.pone.0315536)
Supplement: S3 Fig — (DOCX) [file pone.0315536.s003.docx]

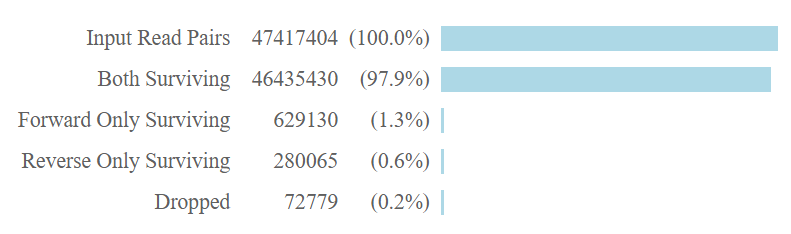


**S1 Fig 3. Output of trimmomatic, a widely used tool for preprocessing high-throughput sequencing data.** The figure illustrates the effectiveness of trimmomatic in filtering and trimming raw sequencing reads to improve data quality before downstream analysis.
