# Supplementary material for: Exploring bioactive compound origins: Profiling gene cluster signatures related to biosynthesis in microbiomes of Sof Umer Cave, Ethiopia
Source: PLoS One. 2025 Mar 6;20(3):e0315536. doi: 10.1371/journal.pone.0315536 (PMC11884727; doi:10.1371/journal.pone.0315536)
Supplement: S4 Fig — (DOCX) [file pone.0315536.s004.docx]

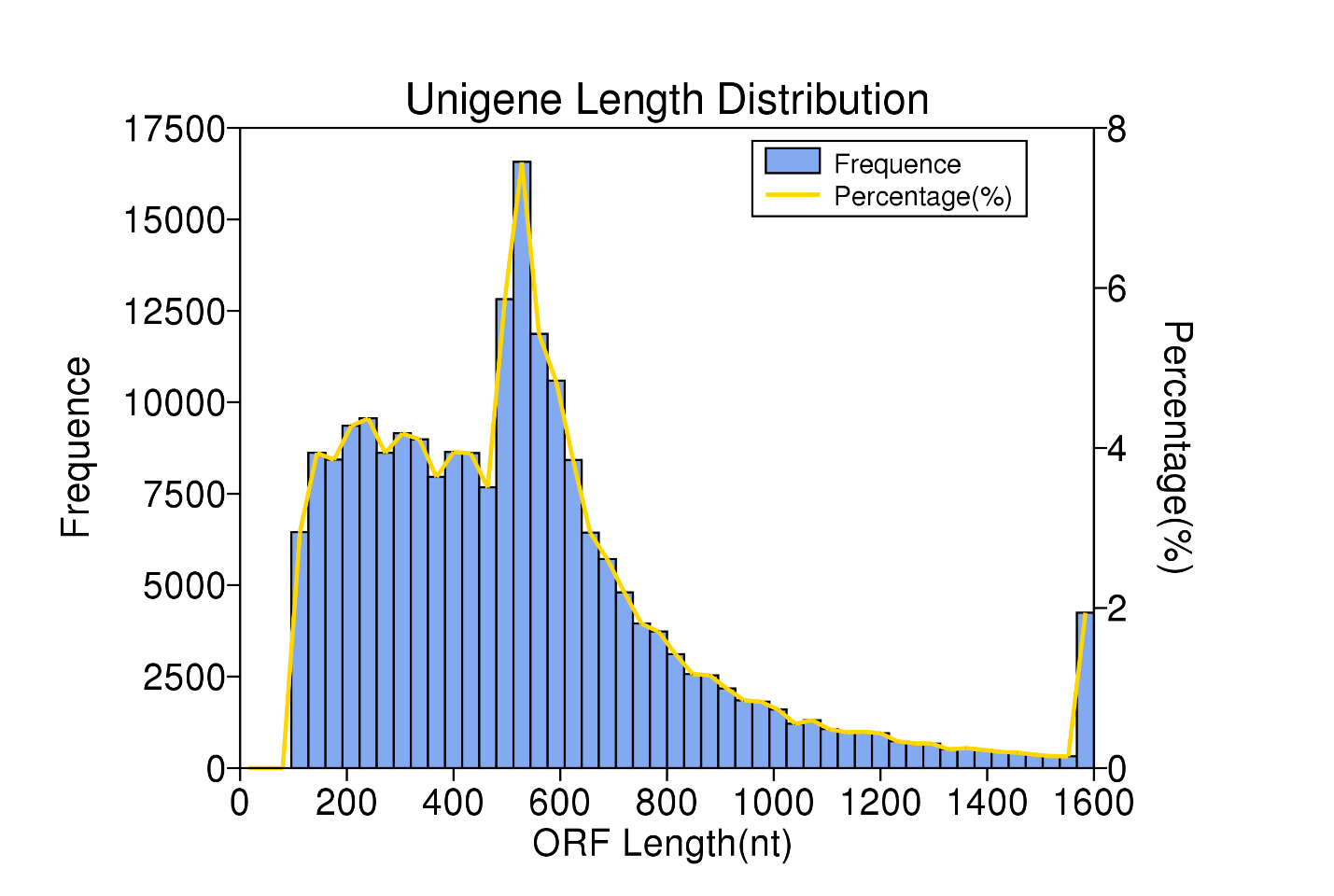


**S1 Fig 4. The scaftig (scaffold) length distribution (scaftigs >500 bp) was used for open reading frame (ORF) prediction.**
