# Supplementary material for: Exploring bioactive compound origins: Profiling gene cluster signatures related to biosynthesis in microbiomes of Sof Umer Cave, Ethiopia
Source: PLoS One. 2025 Mar 6;20(3):e0315536. doi: 10.1371/journal.pone.0315536 (PMC11884727; doi:10.1371/journal.pone.0315536)
Supplement: S5A Fig — (DOCX) [file pone.0315536.s005.docx]

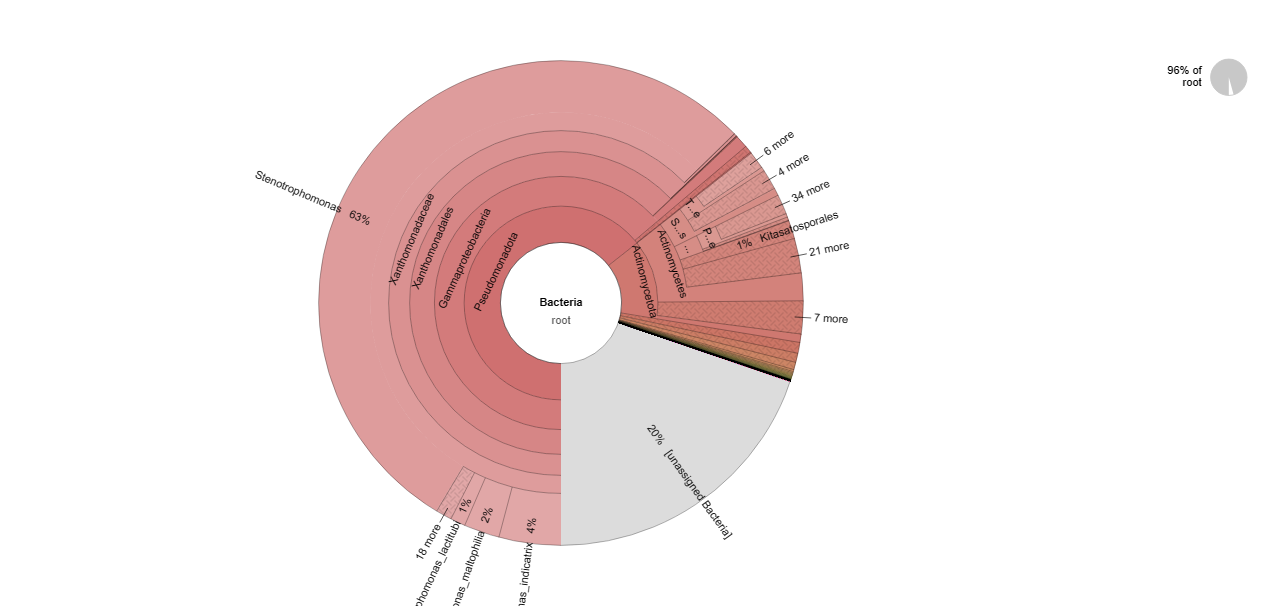


**S1 Fig 5A. Taxonomy and distribution of microbial genomes at the bacterial level via Krona and the Micro-NR database**. Notes: Circles from inside to outside represent different taxa, and the area of the sector represents the respective proportion of different taxa
