# Supplementary material for: Exploring bioactive compound origins: Profiling gene cluster signatures related to biosynthesis in microbiomes of Sof Umer Cave, Ethiopia
Source: PLoS One. 2025 Mar 6;20(3):e0315536. doi: 10.1371/journal.pone.0315536 (PMC11884727; doi:10.1371/journal.pone.0315536)

**S1 Fig 5B. Taxonomies and distribution of microbial genomes in the Actinobacteria phylum using Krona and the Micro-NR database.** Notes: Circles from inside to outside represent different taxa, and the area of the sector represents the respective proportion of different taxa.


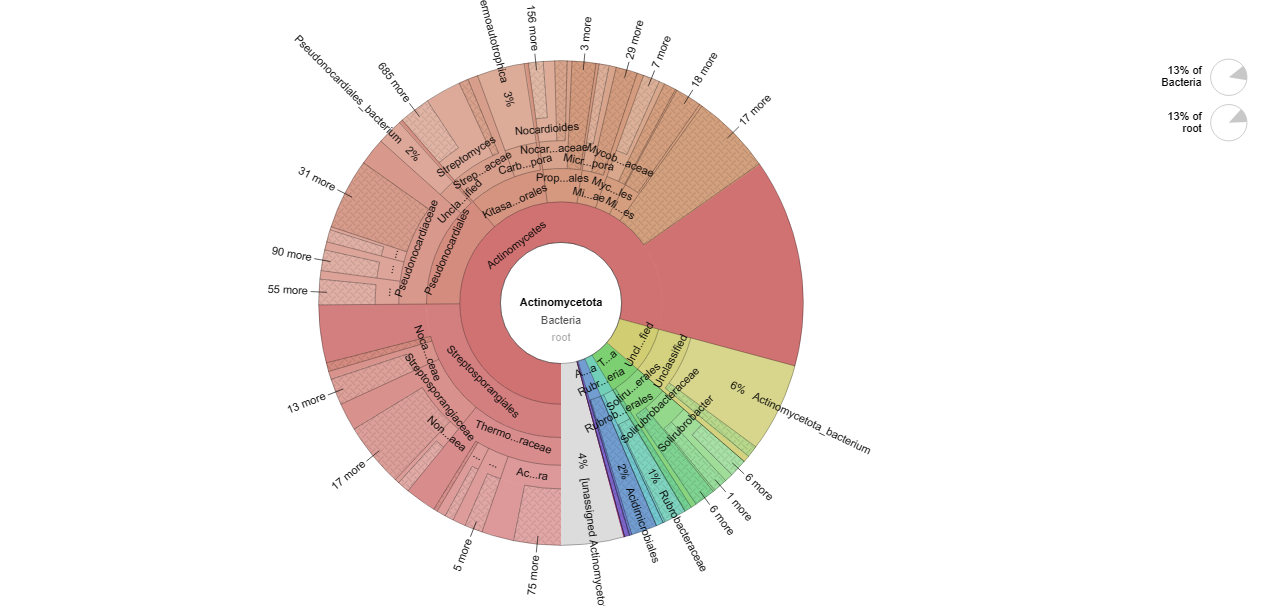

Supplement: S5B Fig — (DOCX) [file pone.0315536.s006.docx]
