# Supplementary material for: Exploring bioactive compound origins: Profiling gene cluster signatures related to biosynthesis in microbiomes of Sof Umer Cave, Ethiopia
Source: PLoS One. 2025 Mar 6;20(3):e0315536. doi: 10.1371/journal.pone.0315536 (PMC11884727; doi:10.1371/journal.pone.0315536)
Supplement: S7 Fig — (DOCX) [file pone.0315536.s008.docx]

k127_105777 - Region 1 - RRE-containing, lassopeptide


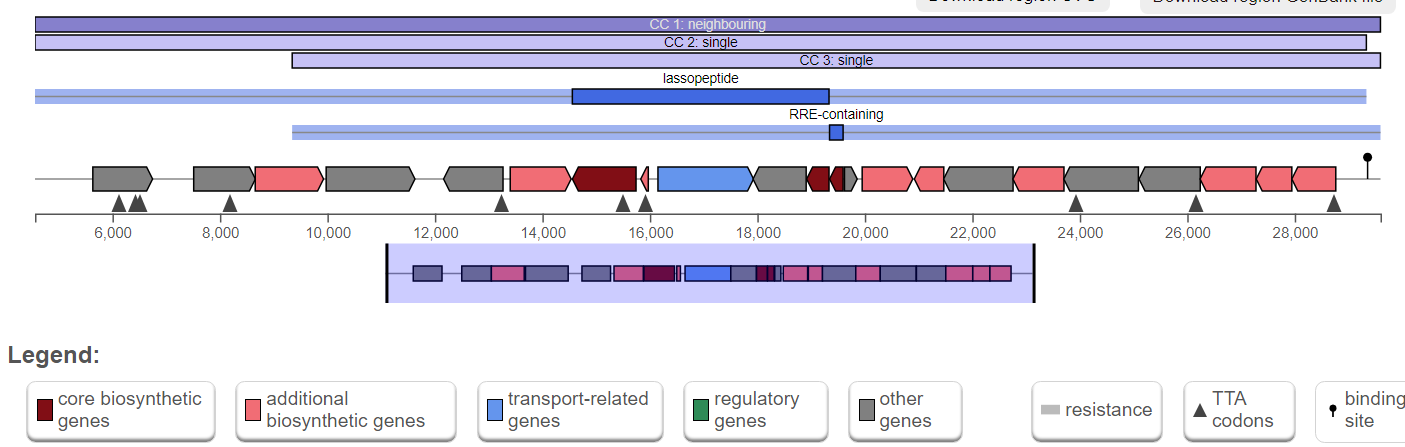

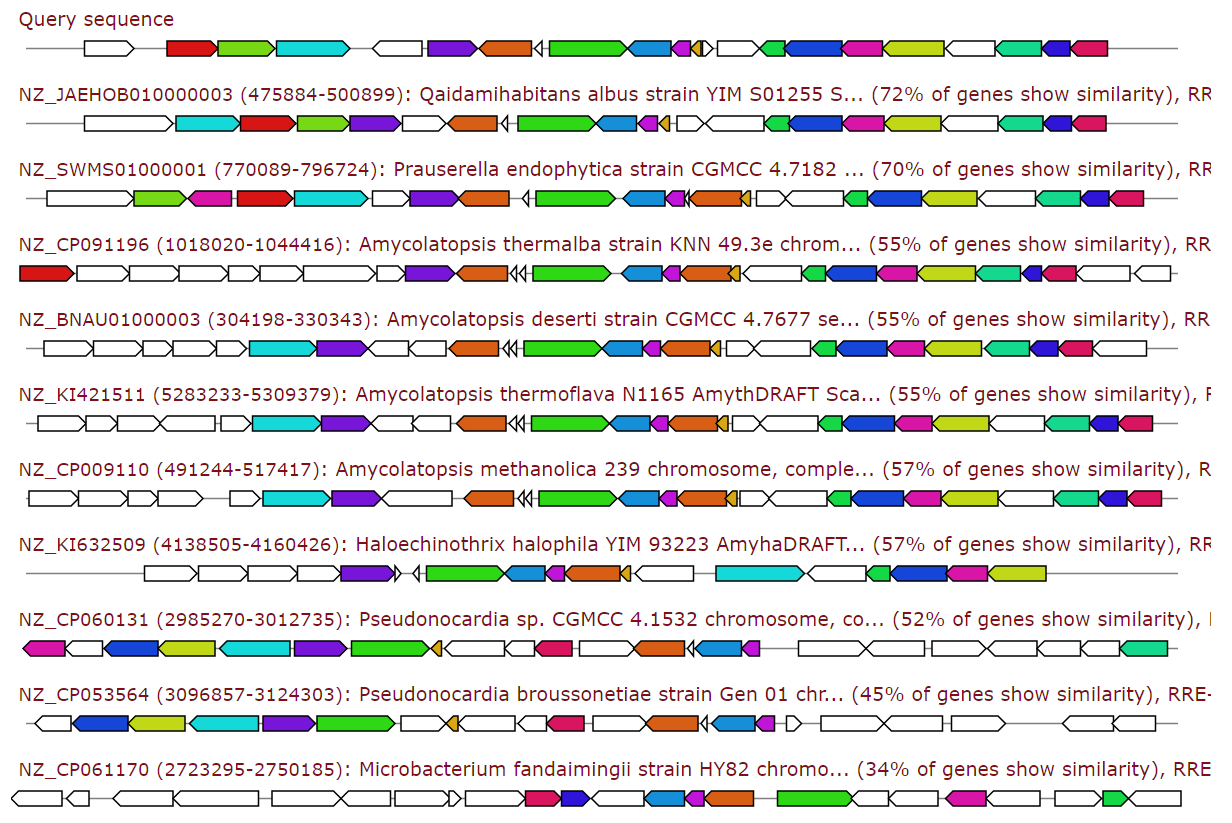


**S1 Fig 7. Selected putative regions of biosynthesis-related gene clusters annotated by antiMASH at 127_57222 - Region 1 - RRE-containing, lassopeptide.**
