# Supplementary material for: Exploring bioactive compound origins: Profiling gene cluster signatures related to biosynthesis in microbiomes of Sof Umer Cave, Ethiopia
Source: PLoS One. 2025 Mar 6;20(3):e0315536. doi: 10.1371/journal.pone.0315536 (PMC11884727; doi:10.1371/journal.pone.0315536)
Supplement: S1 Table — (DOCX) [file pone.0315536.s009.docx]

**S1 Table 1. NanoDrop reads data of metagenomic DNA extracted from the rocks of Sof Umer Cave.**

| No | Sample ID | Nucleic Acid | Unit | A260 (Abs) | A280 (Abs) | 260/280 | Sample Type |
| --- | --- | --- | --- | --- | --- | --- | --- |
| 1 | R1 | 43.7 | ng/µl | 0.294 | 0.172 | 1.91 | DNA |
| 2 | R2 | 37.4 | ng/µl | 0.227 | 0.101 | 2.03 | DNA |
| 3 | R12 | 17.8 | ng/µl | 0.356 | 0.195 | 1.82 | DNA |
| 4 | R6 | 78.5 | ng/µl | 0.21 | 0.125 | 1.80 | DNA |
| 5 | R9 | 377.9 | ng/µl | 7.559 | 3.698 | 2.02 | DNA |
| 6 | R10 | 89.3 | ng/µl | 0.185 | 0.089 | 2.01 | DNA |
