# Supplementary material for: Exploring bioactive compound origins: Profiling gene cluster signatures related to biosynthesis in microbiomes of Sof Umer Cave, Ethiopia
Source: PLoS One. 2025 Mar 6;20(3):e0315536. doi: 10.1371/journal.pone.0315536 (PMC11884727; doi:10.1371/journal.pone.0315536)
Supplement: S2 Table — (DOCX) [file pone.0315536.s010.docx]

**S1 Table 2. Statistics of the gene catalogs.**

| ORFs_NO. | 218,891 |
| --- | --- |
| Integrity_start | 68,428 (31.26%) |
| Integrity_end | 50,728 (23.18%) |
| Integrity_none | 38,924 (17.78%) |
| Integrity_all | 60,811 (27.78%) |
| Total_Len.(Mbp) | 118.29 |
| Average_Len.(bp) | 540.4 |
