# Supplementary material for: Exploring bioactive compound origins: Profiling gene cluster signatures related to biosynthesis in microbiomes of Sof Umer Cave, Ethiopia
Source: PLoS One. 2025 Mar 6;20(3):e0315536. doi: 10.1371/journal.pone.0315536 (PMC11884727; doi:10.1371/journal.pone.0315536)
Supplement: S3 Table — (DOCX) [file pone.0315536.s011.docx]

**S1 Table 3. The secondary metabolite regions were identified via strictness 'relaxed' (truncated to the first 100 record(s).**

| **Region** | **Type** | **From** | **To** | **Most similar known cluster** | | **Similarity** |
| --- | --- | --- | --- | --- | --- | --- |
| [Region 116.1](https://antismash.secondarymetabolites.org/upload/bacteria-9f8c46a2-b8c8-4d57-912a-a124587a83d2/index.html#r116c1) | [ectoine](https://docs.antismash.secondarymetabolites.org/glossary/#ectoine) | 5,131 | 10,510 | [ectoine](https://mibig.secondarymetabolites.org/go/BGC0002052/1) | Other:Ectoine | 75% |
| [Region 152.1](https://antismash.secondarymetabolites.org/upload/bacteria-9f8c46a2-b8c8-4d57-912a-a124587a83d2/index.html#r152c1) | [terpene](https://docs.antismash.secondarymetabolites.org/glossary/#terpene) | 1 | 9,862 | [geosmin](https://mibig.secondarymetabolites.org/go/BGC0000661/1) | Terpene | 100% |
| [Region 398.1](https://antismash.secondarymetabolites.org/upload/bacteria-9f8c46a2-b8c8-4d57-912a-a124587a83d2/index.html#r398c1) | [proteusin](https://docs.antismash.secondarymetabolites.org/glossary/#proteusin),[lanthipeptide-class-ii](https://docs.antismash.secondarymetabolites.org/glossary/#lanthipeptide-class-ii) | 1 | 9,600 |  | | |
| [Region 485.1](https://antismash.secondarymetabolites.org/upload/bacteria-9f8c46a2-b8c8-4d57-912a-a124587a83d2/index.html#r485c1) | [terpene](https://docs.antismash.secondarymetabolites.org/glossary/#terpene) | 10,056 | 30,901 |  | | |
| [Region 506.1](https://antismash.secondarymetabolites.org/upload/bacteria-9f8c46a2-b8c8-4d57-912a-a124587a83d2/index.html#r506c1) | [ectoine](https://docs.antismash.secondarymetabolites.org/glossary/#ectoine) | 45,081 | 55,560 | [ectoine](https://mibig.secondarymetabolites.org/go/BGC0002052/1) | Other:Ectoine | 100% |
| [Region 515.1](https://antismash.secondarymetabolites.org/upload/bacteria-9f8c46a2-b8c8-4d57-912a-a124587a83d2/index.html#r515c1) | [lassopeptide](https://docs.antismash.secondarymetabolites.org/glossary/#lassopeptide) | 1 | 10,632 |  | | |
| [Region 567.1](https://antismash.secondarymetabolites.org/upload/bacteria-9f8c46a2-b8c8-4d57-912a-a124587a83d2/index.html#r567c1) | [ectoine](https://docs.antismash.secondarymetabolites.org/glossary/#ectoine) | 1 | 5,383 | [ectoine](https://mibig.secondarymetabolites.org/go/BGC0002052/1) | Other:Ectoine | 75% |
| [Region 629.1](https://antismash.secondarymetabolites.org/upload/bacteria-9f8c46a2-b8c8-4d57-912a-a124587a83d2/index.html#r629c1) | [terpene](https://docs.antismash.secondarymetabolites.org/glossary/#terpene) | 6,117 | 32,468 | [hopene](https://mibig.secondarymetabolites.org/go/BGC0000663/1) | Terpene | 46% |
| [Region 954.1](https://antismash.secondarymetabolites.org/upload/bacteria-9f8c46a2-b8c8-4d57-912a-a124587a83d2/index.html#r954c1) | [RiPP-like](https://docs.antismash.secondarymetabolites.org/glossary/#ripp-like) | 89,804 | 100,679 |  | | |
| [Region 1252.1](https://antismash.secondarymetabolites.org/upload/bacteria-9f8c46a2-b8c8-4d57-912a-a124587a83d2/index.html#r1252c1) | [lassopeptide](https://docs.antismash.secondarymetabolites.org/glossary/#lassopeptide) | 14,062 | 36,582 |  | | |
| [Region 1337.1](https://antismash.secondarymetabolites.org/upload/bacteria-9f8c46a2-b8c8-4d57-912a-a124587a83d2/index.html#r1337c1) | [lanthipeptide-class-i](https://docs.antismash.secondarymetabolites.org/glossary/#lanthipeptide-class-i) | 1 | 12,349 |  | | |
| [Region 1442.1](https://antismash.secondarymetabolites.org/upload/bacteria-9f8c46a2-b8c8-4d57-912a-a124587a83d2/index.html#r1442c1) | [lassopeptide](https://docs.antismash.secondarymetabolites.org/glossary/#lassopeptide) | 1 | 7,234 | [citrulassin D](https://mibig.secondarymetabolites.org/go/BGC0001550/1) | RiPP | 60% |
| [Region 1547.1](https://antismash.secondarymetabolites.org/upload/bacteria-9f8c46a2-b8c8-4d57-912a-a124587a83d2/index.html#r1547c1) | [lanthipeptide-class-iv](https://docs.antismash.secondarymetabolites.org/glossary/#lanthipeptide-class-iv) | 1 | 9,586 | [labyrinthopeptin A2/labyrinthopeptin A1/labyrinthopeptin A3](https://mibig.secondarymetabolites.org/go/BGC0000519/1) | RiPP:Lanthipeptide | 40% |
| [Region 1685.1](https://antismash.secondarymetabolites.org/upload/bacteria-9f8c46a2-b8c8-4d57-912a-a124587a83d2/index.html#r1685c1) | [T3PKS](https://docs.antismash.secondarymetabolites.org/glossary/#t3pks) | 1 | 14,356 |  | | |
| [Region 1781.1](https://antismash.secondarymetabolites.org/upload/bacteria-9f8c46a2-b8c8-4d57-912a-a124587a83d2/index.html#r1781c1) | [NRPS-like](https://docs.antismash.secondarymetabolites.org/glossary/#nrps-like) | 1 | 18,064 |  | | |
| [Region 1978.1](https://antismash.secondarymetabolites.org/upload/bacteria-9f8c46a2-b8c8-4d57-912a-a124587a83d2/index.html#r1978c1) | [lanthipeptide-class-iv](https://docs.antismash.secondarymetabolites.org/glossary/#lanthipeptide-class-iv) | 1 | 19,313 | [labyrinthopeptin A2/labyrinthopeptin A1/labyrinthopeptin A3](https://mibig.secondarymetabolites.org/go/BGC0000519/1) | RiPP:Lanthipeptide | 40% |
| [Region 1985.1](https://antismash.secondarymetabolites.org/upload/bacteria-9f8c46a2-b8c8-4d57-912a-a124587a83d2/index.html#r1985c1) | [lassopeptide](https://docs.antismash.secondarymetabolites.org/glossary/#lassopeptide),[RRE-containing](https://docs.antismash.secondarymetabolites.org/glossary/#rre-containing) | 4,559 | 29,596 |  | | |
| [Region 2036.1](https://antismash.secondarymetabolites.org/upload/bacteria-9f8c46a2-b8c8-4d57-912a-a124587a83d2/index.html#r2036c1) | [RRE-containing](https://docs.antismash.secondarymetabolites.org/glossary/#rre-containing) | 1 | 13,410 | [citrulassin D](https://mibig.secondarymetabolites.org/go/BGC0001550/1) | RiPP | 40% |
| [Region 2038.1](https://antismash.secondarymetabolites.org/upload/bacteria-9f8c46a2-b8c8-4d57-912a-a124587a83d2/index.html#r2038c1) | [lanthipeptide-class-i](https://docs.antismash.secondarymetabolites.org/glossary/#lanthipeptide-class-i) | 1 | 13,278 | [enduracidin](https://mibig.secondarymetabolites.org/go/BGC0000341/1) | NRP | 6% |
| [Region 2348.1](https://antismash.secondarymetabolites.org/upload/bacteria-9f8c46a2-b8c8-4d57-912a-a124587a83d2/index.html#r2348c1) | [NRP-metallophore](https://docs.antismash.secondarymetabolites.org/glossary/#nrp-metallophore),[NRPS](https://docs.antismash.secondarymetabolites.org/glossary/#nrps) | 1 | 24,345 | [erythrochelin](https://mibig.secondarymetabolites.org/go/BGC0000349/1) | NRP | 85% |
| [Region 2522.1](https://antismash.secondarymetabolites.org/upload/bacteria-9f8c46a2-b8c8-4d57-912a-a124587a83d2/index.html#r2522c1) | [furan](https://docs.antismash.secondarymetabolites.org/glossary/#furan) | 1 | 9,224 | [difficidin](https://mibig.secondarymetabolites.org/go/BGC0000176/1) | Polyketide | 13% |
| [Region 2564.1](https://antismash.secondarymetabolites.org/upload/bacteria-9f8c46a2-b8c8-4d57-912a-a124587a83d2/index.html#r2564c1) | [NRPS-like](https://docs.antismash.secondarymetabolites.org/glossary/#nrps-like) | 1 | 31,956 | [amychelin A/amychelin B](https://mibig.secondarymetabolites.org/go/BGC0002544/1) | NRP | 11% |
| [Region 2731.1](https://antismash.secondarymetabolites.org/upload/bacteria-9f8c46a2-b8c8-4d57-912a-a124587a83d2/index.html#r2731c1) | [terpene](https://docs.antismash.secondarymetabolites.org/glossary/#terpene) | 1,651 | 27,609 | [hopene](https://mibig.secondarymetabolites.org/go/BGC0000663/1) | Terpene | 46% |
| [Region 2808.1](https://antismash.secondarymetabolites.org/upload/bacteria-9f8c46a2-b8c8-4d57-912a-a124587a83d2/index.html#r2808c1) | [lanthipeptide-class-iv](https://docs.antismash.secondarymetabolites.org/glossary/#lanthipeptide-class-iv) | 29,873 | 53,007 | [labyrinthopeptin A2/labyrinthopeptin A1/labyrinthopeptin A3](https://mibig.secondarymetabolites.org/go/BGC0000519/1) | RiPP:Lanthipeptide | 40% |
| [Region 2971.1](https://antismash.secondarymetabolites.org/upload/bacteria-9f8c46a2-b8c8-4d57-912a-a124587a83d2/index.html#r2971c1) | [lanthipeptide-class-i](https://docs.antismash.secondarymetabolites.org/glossary/#lanthipeptide-class-i) | 1 | 8,699 |  | | |
| [Region 3041.1](https://antismash.secondarymetabolites.org/upload/bacteria-9f8c46a2-b8c8-4d57-912a-a124587a83d2/index.html#r3041c1) | [NRPS-like](https://docs.antismash.secondarymetabolites.org/glossary/#nrps-like) | 1 | 14,421 | [ECO-02301](https://mibig.secondarymetabolites.org/go/BGC0000052/1) | Polyketide | 10% |
| [Region 3049.1](https://antismash.secondarymetabolites.org/upload/bacteria-9f8c46a2-b8c8-4d57-912a-a124587a83d2/index.html#r3049c1) | [arylpolyene](https://docs.antismash.secondarymetabolites.org/glossary/#arylpolyene) | 81,269 | 124,877 | [APE Ec](https://mibig.secondarymetabolites.org/go/BGC0000836/1) | Other | 42% |
| [Region 3111.1](https://antismash.secondarymetabolites.org/upload/bacteria-9f8c46a2-b8c8-4d57-912a-a124587a83d2/index.html#r3111c1) | [terpene](https://docs.antismash.secondarymetabolites.org/glossary/#terpene) | 1 | 9,122 | [geosmin](https://mibig.secondarymetabolites.org/go/BGC0000661/1) | Terpene | 100% |
| [Region 3198.1](https://antismash.secondarymetabolites.org/upload/bacteria-9f8c46a2-b8c8-4d57-912a-a124587a83d2/index.html#r3198c1) | [LAP](https://docs.antismash.secondarymetabolites.org/glossary/#lap) | 1 | 7,425 |  | | |
| [Region 3442.1](https://antismash.secondarymetabolites.org/upload/bacteria-9f8c46a2-b8c8-4d57-912a-a124587a83d2/index.html#r3442c1) | [NRPS](https://docs.antismash.secondarymetabolites.org/glossary/#nrps),[NRP-metallophore](https://docs.antismash.secondarymetabolites.org/glossary/#nrp-metallophore) | 1 | 13,482 | [2,3-dihydroxybenzoylserine](https://mibig.secondarymetabolites.org/go/BGC0002689/1) | NRP | 57% |
| [Region 3477.1](https://antismash.secondarymetabolites.org/upload/bacteria-9f8c46a2-b8c8-4d57-912a-a124587a83d2/index.html#r3477c1) | [RRE-containing](https://docs.antismash.secondarymetabolites.org/glossary/#rre-containing) | 1 | 11,656 |  | | |
| [Region 3633.1](https://antismash.secondarymetabolites.org/upload/bacteria-9f8c46a2-b8c8-4d57-912a-a124587a83d2/index.html#r3633c1) | [terpene](https://docs.antismash.secondarymetabolites.org/glossary/#terpene) | 4,721 | 31,068 | [hopene](https://mibig.secondarymetabolites.org/go/BGC0000663/1) | Terpene | 46% |
| [Region 3709.1](https://antismash.secondarymetabolites.org/upload/bacteria-9f8c46a2-b8c8-4d57-912a-a124587a83d2/index.html#r3709c1) | [LAP](https://docs.antismash.secondarymetabolites.org/glossary/#lap) | 1 | 16,637 |  | | |
| [Region 3755.1](https://antismash.secondarymetabolites.org/upload/bacteria-9f8c46a2-b8c8-4d57-912a-a124587a83d2/index.html#r3755c1) | [lanthipeptide-class-i](https://docs.antismash.secondarymetabolites.org/glossary/#lanthipeptide-class-i) | 1 | 14,432 |  | | |
| [Region 3997.1](https://antismash.secondarymetabolites.org/upload/bacteria-9f8c46a2-b8c8-4d57-912a-a124587a83d2/index.html#r3997c1) | [lanthipeptide-class-i](https://docs.antismash.secondarymetabolites.org/glossary/#lanthipeptide-class-i) | 1 | 14,984 | [cebulantin](https://mibig.secondarymetabolites.org/go/BGC0002316/1) | RiPP | 62% |
| [Region 4244.1](https://antismash.secondarymetabolites.org/upload/bacteria-9f8c46a2-b8c8-4d57-912a-a124587a83d2/index.html#r4244c1) | [NRPS-like](https://docs.antismash.secondarymetabolites.org/glossary/#nrps-like) | 12,103 | 39,586 |  | | |
| [Region 4690.1](https://antismash.secondarymetabolites.org/upload/bacteria-9f8c46a2-b8c8-4d57-912a-a124587a83d2/index.html#r4690c1) | [ranthipeptide](https://docs.antismash.secondarymetabolites.org/glossary/#ranthipeptide) | 1 | 11,658 |  | | |
| [Region 4762.1](https://antismash.secondarymetabolites.org/upload/bacteria-9f8c46a2-b8c8-4d57-912a-a124587a83d2/index.html#r4762c1) | [lanthipeptide-class-i](https://docs.antismash.secondarymetabolites.org/glossary/#lanthipeptide-class-i) | 80,055 | 102,594 | [medermycin](https://mibig.secondarymetabolites.org/go/BGC0000245/1) | Polyketide | 5% |
| [Region 5072.1](https://antismash.secondarymetabolites.org/upload/bacteria-9f8c46a2-b8c8-4d57-912a-a124587a83d2/index.html#r5072c1) | [terpene](https://docs.antismash.secondarymetabolites.org/glossary/#terpene) | 12,343 | 33,278 |  | | |
| [Region 5100.1](https://antismash.secondarymetabolites.org/upload/bacteria-9f8c46a2-b8c8-4d57-912a-a124587a83d2/index.html#r5100c1) | [T1PKS](https://docs.antismash.secondarymetabolites.org/glossary/#t1pks),[NRPS](https://docs.antismash.secondarymetabolites.org/glossary/#nrps) | 95,387 | 147,087 | [zwittermicin A](https://mibig.secondarymetabolites.org/go/BGC0001059/1) | NRP+Polyketide | 14% |
| [Region 5432.1](https://antismash.secondarymetabolites.org/upload/bacteria-9f8c46a2-b8c8-4d57-912a-a124587a83d2/index.html#r5432c1) | [ectoine](https://docs.antismash.secondarymetabolites.org/glossary/#ectoine) | 1 | 5,671 | [kosinostatin](https://mibig.secondarymetabolites.org/go/BGC0001073/1) | NRP+Polyketide | 6% |
| [Region 6319.1](https://antismash.secondarymetabolites.org/upload/bacteria-9f8c46a2-b8c8-4d57-912a-a124587a83d2/index.html#r6319c1) | [terpene](https://docs.antismash.secondarymetabolites.org/glossary/#terpene) | 8,096 | 30,066 |  | | |
| [Region 6399.1](https://antismash.secondarymetabolites.org/upload/bacteria-9f8c46a2-b8c8-4d57-912a-a124587a83d2/index.html#r6399c1) | [lanthipeptide-class-i](https://docs.antismash.secondarymetabolites.org/glossary/#lanthipeptide-class-i) | 1 | 13,672 |  | | |
| [Region 6553.1](https://antismash.secondarymetabolites.org/upload/bacteria-9f8c46a2-b8c8-4d57-912a-a124587a83d2/index.html#r6553c1) | [RRE-containing](https://docs.antismash.secondarymetabolites.org/glossary/#rre-containing) | 862 | 23,042 |  | | |
| [Region 6553.2](https://antismash.secondarymetabolites.org/upload/bacteria-9f8c46a2-b8c8-4d57-912a-a124587a83d2/index.html#r6553c2) | [acyl_amino_acids](https://docs.antismash.secondarymetabolites.org/glossary/#acyl_amino_acids) | 49,737 | 112,352 |  | | |
| [Region 6553.3](https://antismash.secondarymetabolites.org/upload/bacteria-9f8c46a2-b8c8-4d57-912a-a124587a83d2/index.html#r6553c3) | [HR-T2PKS](https://docs.antismash.secondarymetabolites.org/glossary/#hr-t2pks) | 219,849 | 262,109 |  |  |  |
