# Supplementary material for: Exploring bioactive compound origins: Profiling gene cluster signatures related to biosynthesis in microbiomes of Sof Umer Cave, Ethiopia
Source: PLoS One. 2025 Mar 6;20(3):e0315536. doi: 10.1371/journal.pone.0315536 (PMC11884727; doi:10.1371/journal.pone.0315536)
Supplement: S4 Table — (DOCX) [file pone.0315536.s012.docx]

**S1 Table 4. Biosynthesis-related gene clusters related to species.**

| Reference | lanthipeptide-class-iv | Similarity score | Type | Compound(s) | Organism |
| --- | --- | --- | --- | --- | --- |
| [BGC0000563](https://mibig.secondarymetabolites.org/repository/BGC0000563/index.html#r1c1) |  | 0.36 | RiPP | Venezuelin | *Streptomyces venezuelae ATCC 10712* |
| [BGC0001226](https://mibig.secondarymetabolites.org/repository/BGC0001226/index.html#r1c1) |  | 0.35 | RiPP | Streptocollin | *Streptomyces collinus Tu 365* |
| [BGC0002337](https://mibig.secondarymetabolites.org/repository/BGC0002337/index.html#r1c1) |  | 0.32 | RiPP | class IV lanthipeptide, SflA | *Streptomyces sp. NRRL S-1022* |
| [BGC0000551](https://mibig.secondarymetabolites.org/repository/BGC0000551/index.html#r1c1) |  | 0.26 | RiPP | SapB | *Streptomyces coelicolor A3(2)* |
| [BGC0002700](https://mibig.secondarymetabolites.org/repository/BGC0002700/index.html#r1c1) |  | 0.25 | RiPP | Bacinapeptin | *Bacillus nakamurai* |
| [BGC0002111](https://mibig.secondarymetabolites.org/repository/BGC0002111/index.html#r1c1) |  | 0.25 | RiPP | andalusicin A, andalusicin B | *Bacillus thuringiensis serovar andalousiensis NRRL B-23139* |
| [BGC0000519](https://mibig.secondarymetabolites.org/repository/BGC0000519/index.html#r1c1) |  | 0.23 | RiPP | labyrinthopeptin A2, labyrinthopeptin A1, labyrinthopeptin A3 | *Actinomadura namibiensis* |
| [BGC0000513](https://mibig.secondarymetabolites.org/repository/BGC0000513/index.html#r1c1) |  | 0.23 | RiPP | Ery-9, Ery-6, Ery-8, Ery-7, Ery-5, Ery-4, Ery-3 | *Saccharopolyspora erythraea NRRL 2338* |
| [BGC0000501](https://mibig.secondarymetabolites.org/repository/BGC0000501/index.html#r1c1) |  | 0.22 | RiPP | Catenulipeptin | *Catenulispora acidiphila DSM 44928* |
| [BGC0000496](https://mibig.secondarymetabolites.org/repository/BGC0000496/index.html#r1c1) |  | 0.22 | RiPP | AmfS | *Streptomyces griseus subsp. griseus NBRC 13350* |
