# Supplementary material for: Exploring bioactive compound origins: Profiling gene cluster signatures related to biosynthesis in microbiomes of Sof Umer Cave, Ethiopia
Source: PLoS One. 2025 Mar 6;20(3):e0315536. doi: 10.1371/journal.pone.0315536 (PMC11884727; doi:10.1371/journal.pone.0315536)
Supplement: S5 Table — (DOCX) [file pone.0315536.s013.docx]

**S1 Table 5. Major types of natural products.**

| Database match id | percent identity | align length | e-value | pathway product | domain class |
| --- | --- | --- | --- | --- | --- |
| k127_103526 | bacit2_C3E | 27 | 88 | 0.066 | [bacitracin](https://npdomainseeker.sdsc.edu/pathway_templates/bacitracin_info.html) |
| k127_106777 | cdaps1_C2_LCL | 55 | 29 | 0.006 | [calcium-dependent antibiotic](https://npdomainseeker.sdsc.edu/pathway_templates/calcium-dependent%20antibiotic_info.html) |
| k127_112466 | cdaps2_C3_LCL | 36 | 67 | 0.010 | [calcium-dependent antibiotic](https://npdomainseeker.sdsc.edu/pathway_templates/calcium-dependent%20antibiotic_info.html) |
| k127_12046 | surfa4_C4E | 26 | 69 | 0.012 | [surfactin](https://npdomainseeker.sdsc.edu/pathway_templates/surfactin_info.html) |
| k127_139162 | act2_C1_start | 39 | 33 | 0.091 | [actinomycin](https://npdomainseeker.sdsc.edu/pathway_templates/actinomycin_info.html) |
| k127_145818 | prist1_C2_LCL | 31 | 100 | 0.023 | [pristinamycin](https://npdomainseeker.sdsc.edu/pathway_templates/pristinamycin_info.html) |
| k127_152912 | tioS_C2_LCL | 28 | 95 | 0.068 | [thiocoraline](https://npdomainseeker.sdsc.edu/pathway_templates/thiocoraline_info.html) |
| k127_192243 | compl1_C1 | 32 | 120 | 0.045 | [complestatin](https://npdomainseeker.sdsc.edu/pathway_templates/complestatin_info.html) |
| k127_197112 | bleom6_C1_LCL | 36 | 81 | 0.041 | [bleomycin](https://npdomainseeker.sdsc.edu/pathway_templates/bleomycin_info.html) |
| k127_197834 | yersi1_C1_cyc | 41 | 73 | 7e-04 | [yersiniabactin](https://npdomainseeker.sdsc.edu/pathway_templates/yersiniabactin_info.html) |
| k127_205117 | prist1_C2_LCL | 42 | 43 | 0.100 | [pristinamycin](https://npdomainseeker.sdsc.edu/pathway_templates/pristinamycin_info.html) |
| k127_267490 | cdaps1_C2_LCL | 31 | 88 | 0.023 | [calcium-dependent antibiotic](https://npdomainseeker.sdsc.edu/pathway_templates/calcium-dependent%20antibiotic_info.html) |
| k127_277979 | compl3_C4E | 33 | 75 | 0.018 | [complestatin](https://npdomainseeker.sdsc.edu/pathway_templates/complestatin_info.html) |
| k127_283813 | cdaps1_C4E | 44 | 27 | 0.075 | [calcium-dependent antibiotic](https://npdomainseeker.sdsc.edu/pathway_templates/calcium-dependent%20antibiotic_info.html) |
| k127_286006 | cdaps1_C1_start | 34 | 82 | 0.042 | [calcium-dependent antibiotic](https://npdomainseeker.sdsc.edu/pathway_templates/calcium-dependent%20antibiotic_info.html) |
| k127_288977 | prist1_C3E | 31 | 61 | 0.041 | [pristinamycin](https://npdomainseeker.sdsc.edu/pathway_templates/pristinamycin_info.html) |
| k127_30821 | cdaps2_C3_LCL | 27 | 109 | 0.028 | [calcium-dependent antibiotic](https://npdomainseeker.sdsc.edu/pathway_templates/calcium-dependent%20antibiotic_info.html) |
| k127_38527 | prist2_C2_LCL | 33 | 39 | 0.081 | [pristinamycin](https://npdomainseeker.sdsc.edu/pathway_templates/pristinamycin_info.html) |
| k127_55307 | syrin1_C4_dual | 42 | 40 | 0.051 | [syringomycin](https://npdomainseeker.sdsc.edu/pathway_templates/syringomycin_info.html) |
| k127_56387 | compl3_C1_DCL | 33 | 93 | 0.001 | [complestatin](https://npdomainseeker.sdsc.edu/pathway_templates/complestatin_info.html) |
| k127_6404 | act2_C3E | 33 | 126 | 0.004 | [actinomycin](https://npdomainseeker.sdsc.edu/pathway_templates/actinomycin_info.html) |
| k127_76395 | cyclom1C5_LCL | 33 | 51 | 0.082 | [cyclomarin](https://npdomainseeker.sdsc.edu/pathway_templates/cyclomarin_info.html) |
| k127_83364 | cdaps1_C1_start | 38 | 42 | 0.045 | [calcium-dependent antibiotic](https://npdomainseeker.sdsc.edu/pathway_templates/calcium-dependent%20antibiotic_info.html) |
| k127_95774 | act3_C1_DCL | 29 | 137 | 0.095 | [actinomycin](https://npdomainseeker.sdsc.edu/pathway_templates/actinomycin_info.html) |
| k127_96060 | bleom8_C1_DCL | 38 | 58 | 0.019 | [bleomycin](https://npdomainseeker.sdsc.edu/pathway_templates/bleomycin_info.html) |
