# Supplementary material for: Exploring bioactive compound origins: Profiling gene cluster signatures related to biosynthesis in microbiomes of Sof Umer Cave, Ethiopia
Source: PLoS One. 2025 Mar 6;20(3):e0315536. doi: 10.1371/journal.pone.0315536 (PMC11884727; doi:10.1371/journal.pone.0315536)
Supplement: Attachment — Submitted filename: Response to Reviewers (PLOS ONE).docx [file pone.0315536.s014.docx]

**Dear Editors:**

We would like to thank you for your invitation to resubmit the revised manuscript. We have addressed each point raised, and we believe that the revisions have improved the quality and clarity of our manuscript.

**Response for Editors**

1. **Additional permits information in the Methods section.**
   - Response: Permission was not required for the collection of sedimentary rock samples. However, we announce the Bale district administration orally because our study was not applied on humans or animals.
2. **Address overlapping text with previous publication (**[**https://doi.org/10.1007/s42452-024-06110-x**](https://doi.org/10.1007/s42452-024-06110-x)**).**
   - Response: We have revised the manuscript to avoid overlap and appropriately cited any relevant material. The text has been rephrased, and the necessary citations have been included.
     - *Changes in the Manuscript*: Specifically, some parts of the methods, results and discussion sections have been revised to avoid self-plagiarism. Please see the highlighted parts.
3. **Competing interests.**
   - Response: We have submitted the competing interests online as per the journal's guidelines.
4. **Captions for the Supporting Information.**
   - Response: We have added captions for all the Supporting Information files of the manuscript and updated the in-text citations accordingly.
     - *Change in Manuscript*: Captions were added to the Supporting Information section.
5. **Provide original uncropped and unadjusted images for blot or gel results.**
6. Response: We have provided original, uncropped and adjusted images of the gel results.

**Response to Reviewer 1**

Dear Reviewer,

We would like to thank you for your valuable comments and suggestions. We have addressed each point raised, and we believe that the revisions have improved the quality and clarity of our manuscript. Below are our responses to each comment, as well as the changes made to the manuscript.

**Reviewer #1:**

1. **Title of the manuscript is acceptable; abstract and introduction are well-written.**
   - Response: Thank you for your positive feedback.
2. **Materials and methods: all the procedures are clearly mentioned.**
   - Response: We appreciate your comments, even though we have made slight modification to this section as per Reviewer 2.
3. **Results and discussion: data is clearly written and well-discussed.**
   - Response: Thank you for your acknowledgment.

We believe these revisions address your concerns and significantly improve the quality of our manuscript. We are confident that the changes we have made have improve our findings and the clarity of our presentation. Please let us know if there are any other areas that require further attention.

Thank you again for your constructive feedback.

**Response to Reviewer 2**

Dear Reviewer,

We would like to thank you for your valuable comments and suggestions. We have addressed each point raised, and we believe that the revisions have improved the quality and clarity of our manuscript. Below are our responses to each comment, as well as the changes made to the manuscript.

**Reviewer #2:**

1. **What is eDNA?**
   - Response: We have added a definition of eDNA in the methodology section to clarify this term for readers who may not be familiar with it.
     - *Change in Manuscript*: “Environmental DNA (eDNA)” refers to DNA collected from environmental samples such as soil.
2. **In Figure 2, "Qualitaty" should be corrected to "Quality."**
   - Response: We have corrected the type error in Figure 2 and ensured clarity and flow in the figure. The revised figure now shows a more logical progression of information, with steps clearly indicated.
     - *Changes in the Manuscript*: Figure 2 has been updated.
3. **Line 119: Amplified through PCR – please provide complete details of the methodology.**
   - Response: We have revised this section to include the complete details of the PCR process, including the reagents, thermal cycling conditions, and specific primers used.
     - *Change in Manuscript*: "eDNA was amplified using PCR with standard PCR protocol"
4. **"An effective concentration" – please mention in units.**
   - Response: We have revised the manuscript to mention the concentration in specific units.
     - *Change in Manuscript*: "50 ng/μL purified environmental DNA was used for ……………………"
5. **Uniformity between Nanodrop and Qubit measurements.**
   - Response: We have ensured consistency in referring to the quantification instrument and clarified where the Nanodrop and Qubit instruments were used.
6. **Data should be deposited in a public repository.**

Response: We have submitted all raw data to the NCBI Sequence Read Archive (SRA) under accession number PRJNA1082540, and a supplementary file was submitted with the manuscript. The data can be accessed at the following link: (<https://www.ncbi.nlm.nih.gov/sra/PRJNA1082540>).

We believe these revisions address your concerns and significantly improve the quality of our manuscript. We are confident that the changes we have made have improve our findings and the clarity of our presentation. Please let us know if there are any other areas that require further attention.

Thank you again for your constructive feedback.
